# Supplementary material for: Predicting visual function by interpreting a neuronal wiring diagram
Source: Nature. 2024 Oct 2;634(8032):113–23. doi: 10.1038/s41586-024-07953-5 (PMC11446822; doi:10.1038/s41586-024-07953-5)
Supplement: Supplementary file 6 — CRF and ERF predictions for individual TmY4 and TmY9 cells. Analogous to Supplementary Data 3, but for TmY target types. Shown are the top four monosynaptic pathways, the strongest pathway passing through each of the top ten intermediary types (ranking from Extended Data Fig. 7), and the trisynaptic pathway Tm1–TmY–Dm3–TmY (see the section entitled Prediction of spatial normalization). [file 41586_2024_7953_MOESM6_ESM.zip › DataS4/TmY9qΓèÑ/720575940621348929.pdf]

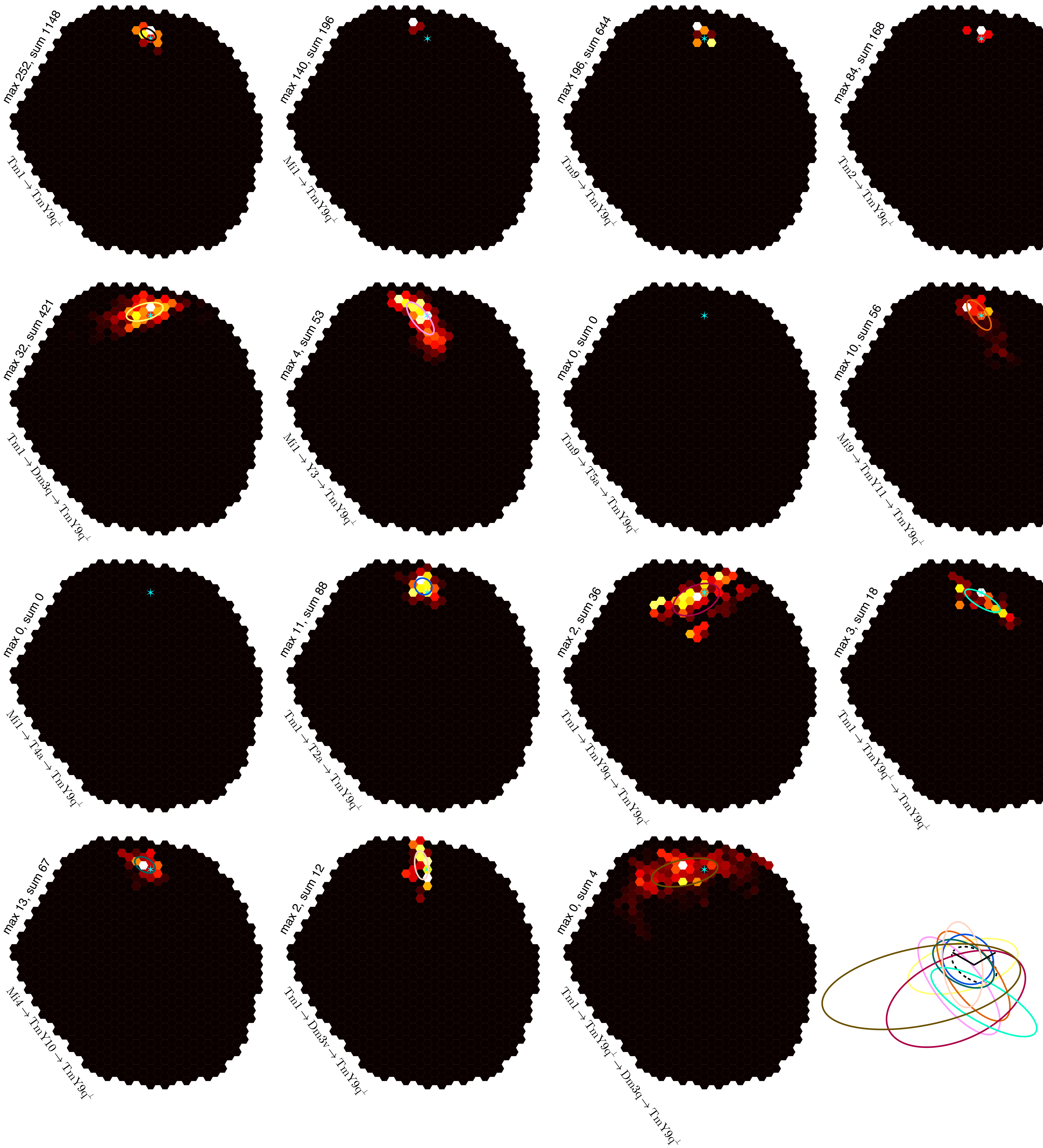

max 252, sum 1148
Tm1 → TmY9q⊥
max 140, sum 196
Mi1 → TmY9q⊥
max 196, sum 644
Tm9 → TmY9q⊥
max 84, sum 168
Tm2 → TmY9q⊥
max 32, sum 421
Tm1 → Dm3q → TmY9q⊥
max 4, sum 53
Mi1 → Y3 → TmY9q⊥
max 0, sum 0
Tm9 → T5a → TmY9q⊥
max 10, sum 56
Mi9 → TmY11 → TmY9q⊥
max 0, sum 0
Mi1 → T4a → TmY9q⊥
max 11, sum 88
Tm1 → T2a → TmY9q⊥
max 2, sum 36
Tm1 → TmY9q → TmY9q⊥
max 3, sum 18
Tm1 → TmY9q⊥ → TmY9q⊥
max 13, sum 67
Mi4 → TmY10 → TmY9q⊥
max 2, sum 12
Tm1 → Dm3v → TmY9q⊥
max 0, sum 4
Tm1 → TmY9q⊥ → Dm3q → TmY9q⊥
